# Gene mention normalization and interaction extraction with context models and sentence motifs

**DOI:** 10.1186/gb-2008-9-s2-s14

**Published:** 2008-09-01

**Authors:** Jörg Hakenberg, Conrad Plake, Loic Royer, Hendrik Strobelt, Ulf Leser, Michael Schroeder

**Affiliations:** 1 Bioinformatics Group, Biotechnological Centre, Technische Universität Dresden, Tatzberg, 01307 Dresden, Germany; 2 Knowledge Management in Bioinformatics, Computer Science Department, Humboldt-Universität zu Berlin, Unter den Linden, 10099 Berlin, Germany; 3 Current affiliation: BioAI Lab, Arizona State University, S Mill Avenue, Tempe/Phoenix, Arizona 85281-8809, USA; 4 Transinsight GmbH, Tatzberg, 01307 Dresden, Germany

## Abstract

**Background:**

The goal of text mining is to make the information conveyed in scientific publications accessible to structured search and automatic analysis. Two important subtasks of text mining are entity mention normalization - to identify biomedical objects in text - and extraction of qualified relationships between those objects. We describe a method for identifying genes and relationships between proteins.

**Results:**

We present solutions to gene mention normalization and extraction of protein-protein interactions. For the first task, we identify genes by using background knowledge on each gene, namely annotations related to function, location, disease, and so on. Our approach currently achieves an f-measure of 86.4% on the BioCreative II gene normalization data. For the extraction of protein-protein interactions, we pursue an approach that builds on classical sequence analysis: motifs derived from multiple sequence alignments. The method achieves an f-measure of 24.4% (micro-average) in the BioCreative II interaction pair subtask.

**Conclusion:**

For gene mention normalization, our approach outperforms strategies that utilize only the matching of genes names against dictionaries, without invoking further knowledge on each gene. Motifs derived from alignments of sentences are successful at identifying protein interactions in text; the approach we present in this report is fully automated and performs similarly to systems that require human intervention at one or more stages.

**Availability:**

Our methods for gene, protein, and species identification, and extraction of protein-protein are available as part of the BioCreative Meta Services (BCMS), see .

## Background

Gene mention normalization and extraction of relationships such as protein interactions from scientific literature are important for three areas. First and foremost, sequence, structure, and other databases are often manually curated based on evidence from literature. With novel high-throughput data generation techniques, however, manual curation is no longer sufficient [1]. Second, interpretation of high-throughput screens such as gene expression or RNA interference screens typically generate large clusters of genes with similar phenotype. Identifying relationships within such clusters such as protein interactions or shared functions, processes, diseases and so on are important for gaining deeper insight. Third, text mining *per se *can serve to cluster genes by phenotype. For example, Lage and coworkers [2] identify candidate genes for diseases by clustering genes based on phenotype terminology extracted from a database with text mining.

### Gene mention normalization and context models

All of these tasks require the identification of genes, terminology, and relationships from text. Finding gene names in text is trivial when a standardized, unambiguous name appears literally in text, but this is rarely the case because authors use different names for a gene, spell names in many ways, or even introduce completely new names. Additionally, gene names are often ambiguous, because they often are abbreviations ('ATF'), reflect a function ('negative factor'), the weight of the protein ('p54'), a disease ('Huntingtin'), a cell type ('CD4'), a person ('Wolf-Hirschhorn'), or other properties that are not unique to the gene. These variants and ambiguities make gene name identification a difficult computational problem. However, biologists reading articles usually do not have a problem identifying genes in text. There are some key differences. Humans do not process millions of articles and gene names, but focus on a limited scope of interest. They do not 'process' articles in isolation, but have background knowledge consisting of detailed knowledge of the genes of interest, including their functions, roles, biological processes, and so on, as well as articles related to the one at hand.

**Figure 1:**
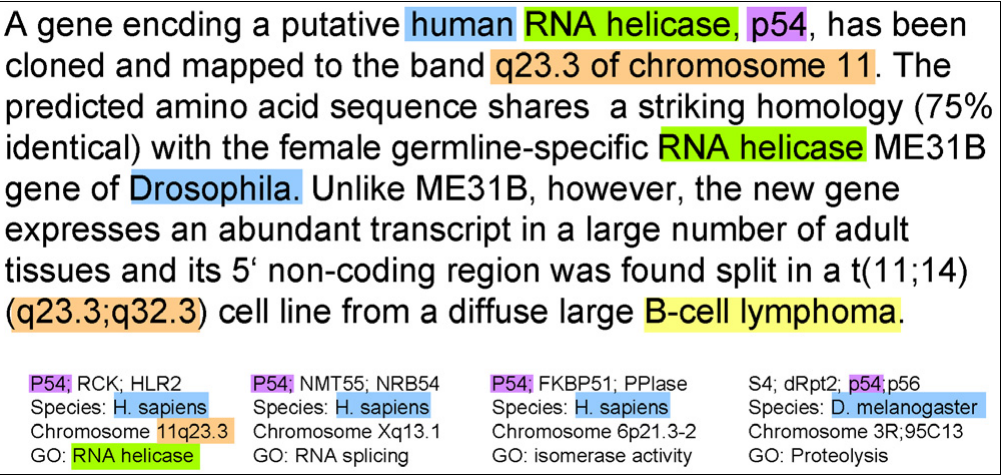
Example for gene mention normalization using context models. Disambiguation in gene mention normalization. Terminology relating to function, location, disease, and so on is explained in the text and defines the textual context, which is matched against potential gene contexts. Although there are four contexts for the gene name 'p54', only one encodes a human RNA helicase and is located on band q23.3 of chromosome 11, as described in the text.

In our approach to gene identification, we try to mimic the human approach. Two key steps are the definition of textual context and gene context. Both contexts capture terms referring to functions, processes, locations, tissue specificities, and so on. Textual context identifies these terms in the article under consideration together with related articles, thus enlarging the text base. Gene context contains the above terms for a gene as documented in high-quality databases. With these context models, gene mention normalization becomes the problem of matching the textual context to the gene context. The two types of context serve opposing purposes. Although textual context extends the text base, thus increasing the potential for high recall, the gene context makes candidate matches unambiguous, thus increasing the potential of high precision. As an example, consider Figure 1. For 'p54' in the text, there are four potentially matching genes. The textual context consists of terms such as 'RNA helicase', 'human' and 'chromosome 11', and best matches the gene context of only one of the four candidate genes.

### Extracting protein-protein interactions with motifs

Gene mention normalization is a prerequisite for the identification of protein interactions in text. Current experimental high-throughput methods for the detection of interactions are still error prone, whereas interactions found in text document high-quality, low-throughput interactions; these carry the potential to improve the overall quality of data. For example, consider the complete set of complexes in the yeast *Saccharomyces cerevisiae *mapped out by Gavin and coworkers [3]. Combining this large-scale dataset with data from a small-scale study reported by King and colleagues [4], investigating nuclear transport, reveals that an interaction between Nup116 of the nuclear pore complex and the Kariopherin Kap95 (a transporter) is not present in the data reported by Gavin and coworkers [3]. Text mining can identify such interactions in text and thus complement experimental large-scale protein interaction studies. Extracting interactions from literature is more difficult than gene mention normalization, because the latter is a first step toward solving the former. If identifying one gene can be achieved with a success rate of 80%, then the rate of correctly extracting a pair of genes is reduced to an expected 64% when we ignore the specific problems of finding real interactions. The rate at which a complete triplet of two genes and their relation can be extracted is thus likely to be well below 50%.

We tackle the problem of extracting interactions by leveraging a well known approach from sequence analysis: motifs derived from multiple sequence alignments. In sequence analysis, functional sites and key residues can be identified by aligning multiple proteins across different species and extracting highly conserved residues. Examples are the NPA-motif in aquaporin or the P-Loop motif for G-proteins. Two key problems in such an approach are the computational complexity and the selection of data. Comparing two sequences of length *n *with dynamic programming is proportional to n^2^. Extending this to *m *sequences increases the complexity to n^m^. To avoid such prohibitive computations, multiple sequence alignments are often approximated by computing pair-wise alignments, and subsequent clustering leads to polynomial complexity [5]. The second problem relates to the choice of sequences. If the sequences are too closely related, then the motifs will be overfitted; if they are too distant, then there will be no motifs.

**Figure 2:**
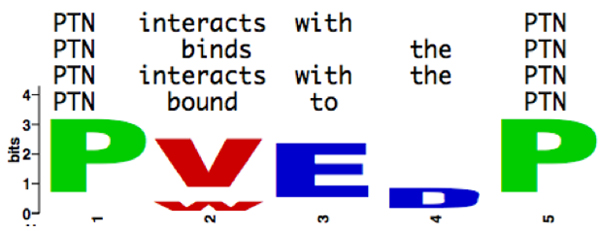
Multiple aligned sentences define a consensus pattern. Logo for a motif derived from multiple sequence alignments that can be applied to sentences of unknown content. PTN represents arbitrary protein names, as does P. V and W are interaction verbs in present and past form, respectively; E is a preposition and D a determiner.

In our approach to protein-protein interaction (PPI) extraction, we create multiple sequence alignments of selected sentences and then derive motifs. Because we are interested in general motifs depicting how authors write about interactions, we replace specific occurrences of concrete protein names by a place holder. As in classical multiple sequence alignments, we approximate the optimum with a clustering-based approach and we carefully select sentences that are not too closely and not too distantly related. An example of a motif is given in Figure 2, in form of a sequence logo. This motif was derived form four different (parts of) sentences and reflects the commonalities between them.

In the following discussion, we give a brief overview of the various steps of the approaches we take to gene mention normalization and extraction of PPIs. We then present quantitative and qualitative results for each approach. We discuss all results and draw conclusions from our work. In the last part of this report we present all of our methods in more detail. To better assess the findings that we present, we recommend reading of the Materials and methods section (below) before the Results and Discussion sections (also below). We present related work where applicable; please also refer to the overview articles on BioCreative II [6-8] for discussions of related work.

### Gene mention normalization

Most systems for gene mention normalization consist of four major building blocks: the acquisition and processing of data (texts and information on genes), the recognition of named entities, and their identification. A similar scheme is presented in the BioCreative II GN task overview article; see the report by Morgan and coworkers [7], where detailed explanations can also be found.

#### Data acquisition

The first stage is the acquisition of knowledge about the entities sought. In the case of gene mention normalization, lexica contain all known names for each gene, and databases contain detailed background knowledge about each gene and links to texts related to each gene. Lexica help in the search for genes based on the pure syntax; background knowledge and text examples help with gathering semantic information about each gene, which can also be searched for.

#### Data processing

In the second stage, data from the first are processed. We analyze known gene names to learn about typical variations (which are currently unknown in the lexicon but might occur in the literature).

#### Entity mention recognition

The third stage handles the actual search for names in a text. From the analysis of known gene names, a search strategy is derived to find these names even if the authors use a nonstandard name.

#### Entity identification

In the fourth stage, all names found in a text are identified. For a gene mention, this refers to finding the database identifier for the gene referenced by the mention. This can be done on a syntactic level (string matching), by searching for the known name from the lexicon that is most similar to the current mention. It can also be done on a semantic level, by comparing information known about each (candidate) gene with the current text.

### Extraction of protein-protein interactions

The simplest systems for extracting PPIs from text look for pairs of proteins that appear together in one sentence. This typically leads to high recall but low precision, because co-occurrence of proteins in one sentence is an almost necessary but not sufficient condition for PPIs. Approaches using machine learning require large, well annotated training corpora, few of which are available for PPIs. Sophisticated natural language processing techniques achieve very good precision, but they often fail when confronted with complex sentences. One approach to finding PPIs is by matching the text against predefined patterns, that is, expressions over words or word stems, pre-recognized tokens, linguistic information, or combinations of those [9-11]. The entire workflow is divided into four stages.

#### Training sample

In the first stage, a training sample is collected from which information on frequent patterns can be drawn. This collection is either done manually, which leads to high-quality data, but is generally time-consuming and results in comparatively small samples. We present a method in this report to collect a very large sample automatically. Note that this sample is independent of any problem-specific training data; instead, it must be computed only once to solve the PPI extraction problem on any corpus.

#### Generating patterns

In the second stage, the sample is used to infer patterns. These patterns cover frequent textual descriptions of PPIs, mostly using sequences of part-of-speech (POS) tags and word lists to generalize observed sentences. We use a mixture of sentence clustering and multiple sentence alignment to derive patterns from sentence examples.

#### Pattern matching

The third stage handles the matching of patterns against new text. Multiple strategies exist, with use of patterns such as regular expressions being the most prominent one. In our approach, we take a bioinformatics approach and use sentence alignment to allow for and quantify inexact matches.

#### Identification of proteins

Stage four identifies the proteins found as partners in PPI. This identification builds on the same ideas described in the previous section on gene mention normalization.

## Results

### Gene mention normalization

We show all quantitative results in Tables 1 to 5. In addition to the initial results on the training and test sets, we show performance of the current system, which contains improvements added after the BioCreative II workshop. For the workshop our best F measure was 81%; the current system achives 86.4% on the test set. Our maximum recall values were between 87.5% and 92.7% (test and training set, respectively). Table 5 shows the impact of using different context types on the performance. Chromosomal locations have the greatest impact on precision (+64.5%), but of course not all abstracts contain such information on all ambiguous genes. To maintain high recall, Gene Ontology (GO) terms are well suited (losing only 9.9% in recall, but gaining 36.6% in precision). The hidden Markov model based context filtering (wrong species and so on) was able to increase the F measure by 45.9%, losing 10.3% in recall.

**Table 1:** Results for gene mention normalization

Short description of the submitted run	Precision	Recall	F measure (%)	True positives (*n*)	False positives (*n*)	False negatives (*n*)
Training set	82.1	81.6	81.8	522	114	118
Training set, no filtering, no disambiguation	20.2	92.7	33.1	593	2,348	47
Test set	78.9	83.3	81.0	654	175	131
Test set, no disambiguation	49.6	87.5	63.3	687	699	98
Test set, unextended lexicon	70.7	72.5	71.6	569	236	216
Test set, current performance	90.7	82.4	86.4	647	66	138

Performance of the gene mention normalization component on the BioCreative II gene normalization sets. Each run includes the extended gene name lexicon, all false-positive filters, and the disambiguation, unless indicated otherwise. Results on the test set reflect official results achieved in the external evaluation; the last row shows the current performance, resulting from improvements added in the aftermath of BioCreative II.

**Table 2:** Performance for gene mention normalization for mouse, yeast, and fruit fly datasets

Short description of the submitted run	Precision	Recall	F measure (%)	True positives (*n*)	False positives (*n*)	False negatives (*n*)
Mouse, training set	86.6	69.2	77.0	322	50	143
Yeast, training set	89.0	84.0	86.4	219	27	42
Fly, training set	87.9	55.6	68.1	124	17	99
Mouse, test set	91.6	72.6	81.0	355	36	149
Yeast, test set	94.9	84.8	89.6	520	28	93
Fly, test set	82.1	69.5	75.3	298	65	131

Current performance of the gene mention normalization component on the BioCreative I gene normalization sets. Each run includes an extended gene name lexicon (based on BioCreative I data and with additional synonyms from EntrezGene), all false positive filters, and the disambiguation.

**Table 3:** Performance of the IPS system on the BioCreative II data

Short description	Precision	Recall	Micro-F	Macro-F
*m *= 2; *gl *= 0.83; *ld *= 3; *ids *= 2; *sr *= a, h, m, y, *	1.8	43.7	3.5	3.1
*m *= 1; *gl *= 0.83; *ld *= 3; *ids *= 1; *sr *= a,*	25.2	23.3	24.2	21.1
*m *= 1; *gl *= 0.75; *ld *= 5; *ids *= 1; *sr *= a,*	22.8	21.6	22.2	19.7

Results for different strategies using the IntAct pattern collection on the BioCreative II test set. *gl*, guide list cut-off; *ids*, number of submitted IDs per protein; IPS, interaction pair subtask; *ld*, maximum length difference; *m*, minimum of identified interactions per pair and article required; *sr*, species resolution (pick first species from abstract [a], pick human [h], mouse [m], yeast [y], or from best scored protein [*], in the given order; see text for explanations on these parameters).

**Table 4:** Performance of PPI extraction on the Spies corpus

Corpus	Short description of the submitted run	Precision	Recall	F measure
Spies	Initial pattern set	85.8	15.2	25.8
Spies	CP, single layer (POS tag including entity)	76.6	47.1	58.3
Spies	CP, multilayer (token, POS tag, stem, entity)	78.7	51.9	62.6
Spies	CP, optimized for precision	+1	-4	60.1
Spies	CP, optimized for recall	-5	+5	63.9

Performance of our approach to protein-protein interaction (PPI) extraction on other external corpora. We also show the influence of using part-of-speech (POS) tags only compared with multilayer alignments, and results for optimization towards a single metric (precision or recall). Note that these evaluations do not require the identification of proteins, as in BioCreative II, so figures are higher in general. CP, consensus patterns resulting from clustering and multiple sentence alignment.

**Table 5:** Impact of different context types on human gene mention normalization

Context type	Precision	Recall	F measure
Baseline: NER only	9.7	91.1	17.5
NER + GeneRifs	50.8	78.3	61.6
NER + GO terms	46.3	81.2	59.0
NER + EntrezGene summaries	49.0	66.7	56.5
NER + diseases	22.7	43.9	29.9
NER + functions	50.8	72.5	59.7
NER + keywords	53.0	53.6	53.3
NER + locations	74.2	14.8	24.7
NER + tissues	39.4	29.1	33.4
NER + immediate context filter (heuristics)	23.5	89.8	37.2
NER + immediate context filter (HMM)	52.9	80.8	63.4
NER + PMIDs	96.2	50.8	66.4

Starting from a baseline configuration (pure recognition of named entities; see text), each context type was evaluated separately. In addition, we present the impact of filtering by the immediate context: excluding genes from wrong species, abbreviations, and similar heuristics, and using an hidden Markov model (HMM) learned from the training data. Using PubMed IDs (PMIDs) curated for each gene (for instance, via GeneRIFs, Gene Ontology [GO] annotation, and UniProt) would be the best way to ensure high precision and F measure, although these data were not used for the BioCreative II evaluation. NER, named entity recognition.

### Protein-protein interactions

On the BioCreative II interaction pair subtask (IPS) test set, our best F measure was 24% (see Table 3); we achieved the highest recall among all participants (43.7%), but with a low precision. These figures include the extraction of the interaction as well as the identification of all participating proteins (two in most cases). Our system yields a maximum recall of 69% for the identification of single proteins, which was the best reported among all participating systems; our highest precision here was 45% and the F measure was 41%. Given that the F measure for gene identification (32,975 human genes) was around 80%, the upper bound for the correct identification of a protein pair would be 64% accordingly (but is actually much lower because the IPS task included more than 300,000 proteins from different species). To measure the performance without the task of protein identification, we tested our system on the Spies corpus [10] (see Table 4). For this task, our best F measure was 63.9%, but the task did not include the identification of proteins.

As described in the Materials and methods section (below), our approach first computes initial patterns from example sentences and then merges them to more general consensus pattern to increase recall. We tested both using the initial patterns directly (and expected good precision but low recall) as well as using the consensus patterns. Starting with an initial pattern set, automatically collected from Medline, we achieve a precision of 85.8% at a recall of 15.2% on Spies. Generalization of the pattern set by multiple sentence alignment to find consensus pattern increases the recall to a maximum of 56.9% at 73.7% precision. Note that prior to our and other systems presented at BioCreative II, approaches proposed for extracting PPIs stopped after the third stage (see section on PPIs under Background, above); predicted evidence for PPIs were reduced to protein names as they appeared in the text, rather than exact identifiers. Thus, results presented on previous benchmarks (such as the Spies corpus) often range between 70% and 90% F measure, but on a much simpler task [9-13].

### Analysis of errors during gene mention normalization

For the gene normalization task, we analyzed the types of error - 175 false positives and 131 false negatives - made on the test set. Table 6 provides a detailed view of errors that cause false negatives or false positives, sorted by error type.

**Table 6:** Sources of errors for the gene mention normalization

*n*	Cause	Evidence or examples
	False negatives	Evidence from abstract/closest lexicon entry

24	Polluting tokens	spectrin betaIV/spectrin beta non-erythrocytic
35	Unrecognized variations (orthographic,	DCoHm/DCOHM
	lexical, structural, morphological)	prothrombin/thrombin
4	Segmentation of name failed	hOBP (IIb)/hOBPIIb
2	Syntactically unrelated	polycomblike/PHD finger protein
66	Removed by filtering step	

	False positives	Examples, with EntrezGene ID

30	Triggered by wrong name boundary	type II *IL-1 receptor*
30	Context filtering (reference to cell etc.)	CD4+
22	TF*IDF filter	five *EGF*-like domains; *ARC *complex
11	Disambiguation picked wrong gene	Nup358 (440872 instead of 5903)
8	Abbreviation resolution failed	Wolf-Hirschhorn syndrome (*WHS*)
4	Wrong species	*Notch1 *(...) murine tissues
2	Overlap of names not recognized	
2	NER missed correct ID	TR2 (8740 instead of 10587)
26	Multiple identifiers for one name	
40	Other	

Analysis of errors that occurred during gene identification, false negatives and false positives, and examples of errors. Words in *italics *are the parts recognized in longer compound names. NER, named entity recognition.

#### False negatives

The most abundant type of false negatives (66 cases) are due to the successive filtering steps and are a consequence of precision-recall tradeoffs. The second most common error was due to unrecognized gene name variations, which account for 35 cases. For example, 'progelatinase A' is in the abstract whereas the most relevant synonym is 'gelatinase A'. Such variations were either lexical, structural, orthographic, or morphologic variations. In 24 cases, additional tokens found in the known synonyms hindered the recognition. For example, 'spectrin betaIV' is in the abstract, but the closest synonym is 'spectrin beta non-erythrocytic'. In most cases, such tokens refer to descriptions, as in the example. (At the time of writing this report, EntrezGene contains the synonym 'betaIV spectrin'.) The segmentation of gene names at strong and weak bonds failed in four cases, one of which involved parentheses; the mention 'hOBP (IIb)' is in the abstract but the closest synonym in the lexicon list was 'hOBPIIb'. Finally, only two gene mentions had no syntactic similarity with any of the synonyms of the valid EntrezGene identifiers, for instance the name 'polycomblike' - a syntactically unrelated name for the PHD finger protein 'PHF'. Given the fact that most false negatives are due to unknown syntactic variations, the recall can be further improved through better coverage of gene name variations. As shown in the 'betaIV spectrin' example, however, the curation of the gene name lexicon also plays an important role; high-coverage, high-quality, and up-to-date sources of gene names are needed, especially because recent abstracts are more likely to contain variations in gene names that are not yet listed in databases.

One way to improve the coverage of variation rules is to learn them automatically from the synonyms in the lexicon. An example for which this could have helped is '5HT4sR' (3360) -where 's' is absent from all abbreviations in the lexicon list (for instance, '5-HT4R') - but which could have been learned from the compound name '5-hydroxytryptamine (serotonin) receptor 4'. We postulate that such structural, morphologic, orthographic, and lexical variations could be learned automatically from the analysis of dictionaries [14].

#### False positives

Most false positives were strict in the sense that they were wrong recognitions, regions of text not referring to a gene. Many wrong recognitions (30 cases) were due to parts of a gene mention, such as 'IL-1 receptor' being recognized instead of the whole mention 'type II IL-1 receptor'. Also, the context filtering rules did not cover enough cases (another 30 false positives). For example, the mention '*ARC *complex' refers to a complex and not to the '*A*poptosis *R*epressor with *C*ARD domain' gene. Another example is 'inhibitors of *PI 3-kinase*', which does not refer to the kinase itself but to its inhibitors in general.

In 26 cases, multiple identifiers were kept for the same mention; this was due to the fact that all identifiers had high and similar scores and thus, instead of having both a false positive and a false negative, it was deemed better to keep several candidates. This assumption held only for the training dataset, though, producing much fewer false positives and resulting in more true positives than on the test set. In 22 cases, false positives such as *TNF *were not filtered by TF*IDF scoring. In 11 cases the disambiguation failed to pick the right identifier, such as the mention 'Nup358', for which a 'GRIP domain containing' gene (440872) was chosen instead of the 'RAN binding protein 2' (5903). The resolution of abbreviations failed in eight cases; in the mention 'Wolf-Hirschhorn syndrome (WHS)', 'WHS' was confused with the gene 'Wolf-Hirschhorn syndrome candidate 1' (7468), whereas the abstract discusses the candidate 2 (7469). This error depicts the difficulty of distinguishing a gene mention from diseases, domains, complexes, and other entities to which they are biologically related. Finally, in two cases, the recognition missed to assign the right identifier altogether (but assigned other candidate IDs).

From this analysis we, learn that the strength of our system was in tackling false positives from a semantical point of view; few mistakes were made in choosing the right identifier from among wrong candidates. Instead, many false positives are due to wrongly delimited matches or to mentions of other entities confused for genes, most of which can be detected at the syntactical level using hints such as keywords ('complex', 'inhibitors', 'superfamily') before and after a candidate mention. Thus, precision can be further improved by a better syntactical analysis of the text, and of the contextual clues around candidate gene mentions.

### Analysis of errors in extracting protein-protein interactions

Because of the nature of the BioCreative II IPS benchmark, error analysis proved not as easy as on the BioCreative II gene normalization data. The benchmark consists of a large number of full text articles, and annotations are available only externally in the form 'PubMed-ID|UniProt-ID|UniProt-ID', thus not pointing out exact evidence for the interactions. Assessing each false positive/negative thus means reading each full-text article and identifying proteins by hand. Here, we describe the main sources of errors qualitatively rather than quantifying them.

We noticed that our patterns often are not suited to fit large enumerations, like 'A interacts with B, C, and D'. There are two reasons for this type of error. First, sentences fitting the given example were not contained within the initial set of core phrases, and thus the subsequent clustering and multiple sentence alignment failed to generate a pattern matching the example. Second, fitting sentences were contained in the initial set, but the annotation for PPIs that came from known IntAct pairs did not include all pairs in the sentence. For instance, we could have a core phrase with an annotated interaction 'A/C' and one with the interaction 'A/D', but none with 'A/B'. Parsing of sentences to appreciate that B, C, and D are part of the same enumeration, and thus are all related to A in the same way, would be a way to tackle this problem. Another option would be to include grammars to deal with list of proteins ('PTN → PTN|PTN and PTN').

Many of our patterns are subpatterns of others. We saw that whenever a larger pattern matches the given sentence, the shorter pattern should not be used at all. This resulted in errors such as in the example 'A interacts with B and C interacts with D', where a short pattern 'PTN interacts with PTN and PTN' will return a false-positive interaction between A and C.

A particular source for false positives was dangling text and bibliographies. Although the predicted interaction might be correct, they were not contained in the main part of the current article and thus false. In the same manner, we found that many false positives result from the interactions discussed, for instance in introductory sections of an article. These were not regarded as main statements made by the authors of that article, but mere background information, and thus not included in the benchmark. However, a simple filtering by the number of occurrences of each single predicted interaction (background information: only once; main results of the current article: multiple times) should help to solve this issue.

Some errors, mainly affecting the protein name recognition, resulted from poor PDF to ASCII conversion of articles. This was necessary to make each article machine readable, but led to names lacking Greek letters, or superscripts and subscripts. In particular, Greek letters are quite often parts of protein names. When these are missing from names of members of protein families ('eIFGα' through 'eIFGκ' all become reduced to 'eIFG'), recognition and, in particular, identification are much more difficult if not impossible.

## Discussion

### Gene mention normalization

There are some other systems that also use gene context modeling information on each gene drawn from different sources. For example, Fundel and coworkers [15] and Xu and colleagues [16] search for UMLS concepts assigned to each gene in the text surrounding ambiguous mentions. They report comparable performances, and it remains to be seen how our system could benefit from including UMLS terms. Instead of comparing exhaustive descriptions for diseases or molecular functions (such as from UniProt) to texts, recognition of plain terms might lead to more precise mapping of gene contexts to texts.

We calculated the performance of our system on BioCreative I Task 1B data, where the task was to identify genes from yeast, mouse, and fruit fly, on three separate datasets. Table 2 shows the results for these tasks. Performance was about 4% better for yeast genes (F1 86.4% and 89.6% on training and test set, respectively), and 4% worse for mouse genes (77% and 81%), as compared with our results on the human dataset. The F measure on the fruit fly data (68.1% and 75.3%) was mainly affected by the low recall of these genes (55.6% and 69.5%). The respective top scoring systems for each of these species in BioCreative I achieved F measures of 92, 79, and 82% on the three test datasets [17]. Gene names from yeast are less ambiguous than names from human; on average there are 1.01 genes per name for yeast and 1.03 for human, and so the disambiguation task is somewhat easier for yeast. The same argument also holds for fruit fly data, for which there are 1.13 genes per name, making the disambiguation much harder (mouse: 1.02). One other explanation for the worse performance on mouse and fruit fly data could be an overfitting of the named entity recognition component towards human gene names. Also, 'in-general-mentions', not referring to any particular species, were treated as correct mentions according to the current annotation guideline. BioCreative I data were slightly less ambiguous for mentions of genes from different organisms (data not shown). An indication for overfitting to the training data could also be the vast difference of performance on training and test sets (up to 7% in F measure on BioCreative I, as compared with only 0.8% on the BioCreative II data).

Assuming perfect named entity recognition, which does not miss any mention nor find false positive names (for instance, wrong species), but perhaps assigns multiple candidate IDs to a name, we noted an increase in performance by more than 10%. This scenario is close to the study conducted by Xu and coworkers [16], who reported a precision of 92.2% at 93.8% coverage on a simplified subset of the BioCreative II gene normalization data [16] (114/262 abstracts), and leads to comparable results. The search for named entities, in our approach based on inexact matching against a dictionary, could also draw from other approaches proposed for named entity recognition. These often build on machine learning techniques, such as sequence models, to also spot previously unseen named entities (see overview article on the BioCreative II gene mention task in this supplement [18]).

### Protein-protein interactions

There are several approaches to the extraction of PPIs from text. A large number of systems uses hand-crafted pattern sets, such as [9] and [19]. Although these systems may reach very good precision, the effort necessary to obtain at least acceptable recall on the sentence level is very high. Another class of systems relies on pure machine learning and casts the extraction task as one of classifying a sentence (or an abstract); an example is the PreBind system [20]. However, these systems usually can only point to a sentence describing an interaction, but not the type and partners of it. The system most similar to our approach was described in [10]. The method described there learns patterns for information extraction from an annotated corpus, which is very different from our method. Noteably, our results on the corpus used in this paper are only marginally worse than those of the authors, despite the fact that we do not use any training data. This hints toward the high robustness of our ideas. A simpler but comparable approach to extract mentions of gene and protein mutations was presented in [21]. Finally, the general idea of sentence alignment in a method similar to the alignment of sequences has already been used in different fields of linguistics, such as in paraphrasing [22].

Our method for extraction of PPIs works completely independent from the training corpus, which we did not use at any stage. Thus, we intrinsically exclude any risk of overfitting, and believe that our approach should work equally well for related extraction problems, such as finding associations of proteins with diseases.

We tried to score potential PPIs by comparing the GO annotations of both partners with each other. A model for such a comparison can be learned from the provided training data. We used all annotated PPIs as positive examples; whenever two proteins occur in the same publication, but were not annotated as interacting, we took those as a negative example. In theory, such a model should be able to tell whether two proteins are similar enough (concerning their annotation) to be interacting, but not too similar. For instance, two spatially interacting proteins could be involved in the same biologic process and thus share this annotation, but in terms of function one could be a receptor and the other a ligand; hence, these annotations differ slightly. We found the predictive power to be too weak, however, mainly caused by the lack of proper negative examples. Schlicker and coworkers [23] present a similar approach to score interactions in large-scale datasets and subsequently identify likely false positives [23].

Sentence parsing proved to be more precise than pattern matching approaches, as used by our system, for instance shown in [12,13]. During the past few years, sufficiently well performing tools for sentence parsing and resolving the dependency structure have been proposed, and also tested on biomedical corpora [24]. Saetre and coworkers [25] presented a system that built on patterns over dependency structures instead of mere POS tags and surface information. Using some of the stages of our approach, it is possible to extract large samples to also learn these types of patterns.

## Conclusion

We found that comparing a gene's known context model with the text it is potentially discussed in improves the disambiguation of candidates significantly compared with pure syntactic approaches (that use string matching against a dictionary). In particular, chromosomal locations, molecular functions, and biologic processes known for a gene or its products increase the precision. Also, the comparison of GeneRIFs with the text at hand helps to select the right gene from among multiple candidates.

Future work will cover automated analysis of gene name lexica to identify common variation patterns. These can either be transformed into rules to generate synonyms, or included in the matching strategy. Such an approach could also be applied to other domains, such as disease or drug names, or GO terms. We plan to study the forward annotation of genes using nearest candidate selection based on associated texts (for example, noisy data) or z scores of the disambiguation component in more detail. Thus far, the predictive power of both approaches was too low. A string matching of known synonyms for each such predicted gene following the semantic matching might help to increase performance; the string matching could be less strict than when used as a single component.

In addition to the gene normalization approach, we also presented a fully automated system to find and refine language patterns (sentence motifs) for PPIs. These patterns can be aligned to arbitrary text to extract new PPIs, or they can be used to learn pattern sets as shown by Hao and colleagues [10]. Our approach is applicable to other domains, as long as high-quality databases covering relationships of interest (for instance, gene-disease associations) are available. Current work on the IPS task mainly is concerned with speeding up the processes of computing consensus patterns and applying them to new text. Our method for both steps is based on pair-wise and multiple sentence alignment; both processes are computationally complex. In addition to estimating likelihoods based on pattern (usage) frequencies, we are also studying different strategies to recognize patterns in text, other than alignment. One strategy in particular is direct memory access parsing (DMAP), where we transform patterns into grammars. These could, for instance, handle enumerations of proteins properly ('protein → protein | protein-list'). A publicly available framework for DMAP is OpenDMAP [26]. Finally, we believe that the next generation of PPI extraction tools will also include interactions that are described across sentences - a frequent and common linguistic phenomenon that is not handled by any PPI extraction tool that we are aware of.

## Materials and methods

This section presents our methods for each of the aforementioned stages of gene mention normalization and PPIs. Gene mention normalization then forms a building block for the identification of protein pairs.

### Gene mention normalization

#### Data acquisition

##### Dictionaries

Our dictionaries were based on the lexica provided with the BioCreative II datasets. For gene name identification, this lexicon consisted of 32,975 human genes from EntrezGene. For protein names, there were 323,547 entries from SwissProt. To ensure higher recall, we added more known synonyms to each gene/protein. We found additional gene names in EntrezGene's 'Other designators' field, and also retrieved all names for each protein referenced in EntrezGene (by UniProt ID). For the protein identification (as a subtask of IPS), we found that the same protein often had slight spelling variations concerning 'standard' names across different species. For instance, compare the names 'Hoxb4' (human) and 'HOXB4' (mouse). Authors use either variation to refer to one of these proteins. We thus combined all similar names (no white spaces, case insensitive, no symbols) into sets of IDs.

##### Background knowledge

We collected background knowledge from EntrezGene, UniProt, and GO annotation (GOA) for each of the 32,975 genes (EntrezGene: summary, GO terms, GeneRIFs, chromosomal location, interaction partners; UniProt: diseases, keywords, functions, protein mutations, protein length, protein domains, GO terms, interaction partners, tissue specificity; GOA: GO terms).

#### Data processing

After extending the dictionaries with additional synonyms for each gene/protein in the way described, we transformed every name into a regular expression to match orthographical, morphological, and some structural variations likely to be used by authors. To generate the regular expressions, we grouped all synonyms from the lexicon into either of four categories: database identifiers, abbreviations and acronyms, compound names, and unlikely gene names. We treated any instance of these four groups differently with respect to the way in which we generated regular expressions. We filtered out unlikely names ('AA', "ORF has no N-terminal 'Met', it may be non-functional", single letters, numbers) entirely and concentrated on the other three groups instead.

In the case of database identifiers, we searched for them using manually encoded regular expressions. A match triggered an immediate identification of the referenced gene/protein; if it was contained within the gene lexicon, then no further processing was required. To generate regular expressions for abbreviations and acronyms, we segmented each such name into components for which we observed and thus could express frequent variations. This segmentation was triggered by strong and weak bonds within a name. White spaces and hyphens are strong bonds, weak bonds occur for every other change in the flow of characters (between upper and lower case letters; between letters and digits). We introduced weak bonds also for every first and last letter in a sequence of letters. For each segment, we generated potential variations based on observations in the lexicon list and training data. In general, variations allowed for changes in the surface pattern of the following: letter sequences such as MYD, Myd, myd, and MyD; switches between Roman and Arabic numbering, such as 2, ii, and II; single letters for Greek characters such as α, a, A, alpha; and special single letters such as R, r, or receptor and L, LG, l, or ligand.

Possible variations for each segment were combined into a regular expression; all expressions for all segments defined an expression for the whole abbreviation, with any kind of gap in between. Examples are as follows: HER2 = {HER, HeR, Her, her} [-]? {2, ii, II}; IFN-gamma = {IFN, Ifn, IfN, ifn} [-]? {g, G, gamma}; and MYD88 = {MYD, MyD, Myd, myd} [-]? {88}.

In addition, abbreviations of human gene names often feature an additional 'h' at the beginning, so this was added as optional to every abbreviation. We segmented compound names at white spaces. Every segment (a token) was then treated similar to abbreviations. Tokens that resembled English words (initial upper or lower case, then all lower case letters) occurred in fewer variations (all lower or initial upper). This ultimately led to a regular expression for the whole compound name. Some tokens in a compound name often are left out in free text, such as 'protein' or 'domain'. We thus encoded all these as optional in the regular expression.

We removed names from regular expressions when they matched one of 7,700 stop words from the BNC frequency list [27]. Hand-crafted rules also removed matches like 'or 45' and 'and 1', triggered by too loose regular expressions for the gene names 'Or45' and 'And-1', respectively. For some names, like 'protein 1' or 'antigen 2', we did not generate regular expressions that allowed for variability, but required exact matches.

#### Entity mention recognition

We preprocessed all texts to find ranges and enumerations of (potential) gene names. Each such range was replaced with a list of all expanded names. For example, occurrences such as 'hHR23A and -B' were replaced with 'hHR23A and hHR23B', and 'freac-1 to freac-3' was replaced with 'freac-1, freac-2, freac-3'. We combined consecutive and overlapping evidence; overlapping names such as 'IL-1 receptor' ('IL-1' and ;IL-1 receptor are both valid gene names) and abbreviations with definitions such as 'vesicular monoamine transporter (VMAT)' were treated as a single item of evidence. In both cases, we retained all candidate IDs for the longer form if the two sets did not overlap, or built the union if there was an overlap in IDs.

We encoded all regular expressions for all gene names together in a single finite state automaton. This automaton had end states for every potential match, and each end state accumulated all corresponding EntrezGene IDs (UniProt IDs for the IPS task). A match with the automaton then triggered a recognition with an initial set of candidate IDs for further filtering and disambiguation. Matches required word boundaries around each name ({blank, (/} before the match; and {blank, . : ; /) + - '(+'} after the match).

#### Identification of entities

Identification of genes was done in two steps. First, we tried to filter out false positive names -that is, names not referring to human genes - according to some statistics and heuristics, which we explain in the following text. The heuristics invoked the immediate context of a name, which might contain evidence that the name refers to a different species (not human or mammal), that it refers to a disease, that it is an unspecific mention (of a protein family), or that it refers to a common English word. The second step disambiguated remaining names by ranking all genes that were candidates. Ranking was performed by comparing background information on each of the candidate genes with the current text, scoring different types of background information, and picking the gene that most likely is discussed in the text.

#### Filtering of names

After the initial recognition of potential gene names, we proceeded with filtering out or trying to identify each name. We passed the annotated texts through several filters to reduce the number of false positives and to find the correct dictionary entry. These filters checked for abbreviations defined in the same abstract and references to wrong species, cell lines, or diseases.

##### Filtering by immediate context

To distinguish between names referring to genes and names referring to other concepts (for instance, protein families or protein domains), we analyzed their immediate context - that is, the two words adjacent to the current name, skipping over the most frequent stop words. To build a statistical model, we annotated the training data from BioCreative I and II with dictionaries for each species (mouse, yeast, fruit fly, and human). All matches were then separated into the two classes 'gene names' and 'other concepts' by comparison with the gold standards. We then used these two sets to build a hidden Markov model for each class. Each model contained three states (left, middle/name, and right), with according token emissions. We tested these models against the human test set and found the log-likelihood ratio to be statistically significant (*P *< 0.0003) to distinguish between human genes and other concepts.

##### Abbreviations

If a gene name was found as a single token inside brackets, then we treated it as an abbreviation and tried to resolve it to its long form in the preceding text. In case a long form was found and it did not contain, for example, 'protein', 'gene' or 'factor', or ended with '*ase', or was not recognized as a gene name itself, we treated the short form as a nongene abbreviation.

##### Filtering for wrong species, cell lines, and disease names

We also checked for unwanted references (different species, cell line) and gene names that overlap with disease names. In general, we removed every gene name that had an adjacent species different from human, was followed by a references to a cell (line), or resembled a disease name. We recovered some of such filtered names by keeping genes that mention a different species, but also human (or general references to mammals) somewhere in the same sentence; we kept disease names when they were used as reference to a chromosomal locus. Table 7 shows some examples for heuristic rules to find such occurrences.

**Table 7:** Filtering rules for species, direct references, and chromosomal locations

	Species
-	*non-human-species *<candidate name>
+	*human *and *nonhuman-species *<candidate name>
-	<candidate name> {(, ','} {a, an, the} *not-human-species*
+	<candidate name> {(, ','} {a, an, the} human
+	*human *<candidate name> {(, ','} {a, an, the}
	Direct mentions, cell lines, chromosomal loci
+	<candidate name> {gene, protein}
-	<candidate name> {cell(s), culture(s)}
+	{locus, loci, location, chromosome, chromosomal, gene * associated}

Examples for heuristic rules to filter out candidate names when they appear to refer to some other concept (gene from another species, cell line, disease locus). '<candidate name>' refers to the occurrence of the potential gene name under consideration. Keep (+) or remove (-) a candidate name when the sentence contains the pattern ('+' rules have preference). '*human*' includes references to mammals.

##### Filtering unspecific names

We cross-checked each name against a predefined regular expression that matches unspecific names. It consists of unspecific tokens ('isoform', 'protein', 'liver', and 'membrane'), and we allowed for any permutation and number of those (see Additional data files).

#### Disambiguation by candidate ranking

The final component for gene name identification disambiguated each polysemous name. We compared background knowledge available for each gene (gene context) with the current text and picked the gene whose context best fitted the current text. We collected external knowledge from EntrezGene, UniProt, and GOA for each of the 32,975 genes (EntrezGene: summary, GO terms, GeneRIFs, chromosomal location, interaction partners; UniProt: diseases, keywords, functions, protein mutations, protein length, protein domains, GO terms, interaction partners, tissue specificity; GOA: GO terms).

To calculate the similarity based on GO terms, we searched for GO terms in the current abstract and compared them with the set of GO terms assigned to each gene candidate. For each potential tuple taken from the two sets (text and gene annotation), we calculated a distance of the terms in the ontology tree. These distances yielded a similarity measure for two terms, even if they did not belong to the same sub-branch or were immediate parents/children of each other. The distance took into account the shortest path via the lowest common ancestors, as well as the depth of this lowest common ancestor in the overall hierarchy (comparable to the report by Schlicker and coworkers [28]). The distances for the closest terms from each set then defined a similarity between the gene and the text.

We compared the other annotations from each gene's context model according to their type (terms or full texts). For terms (such as keywords), we calculated the fraction of terms occurring in the abstracts among all terms. For texts (for instance, descriptions of a gene's implications in diseases), we calculated the cosine distance of both bag-of-word representations (with term frequencies, TF, as weights) and the normalized overlap (fraction of tokens from the disease description that also occur in the abstract). We excluded 154 stop words and other, nondiscriminative tokens such as 'protein', 'observe', and 'detected'. All such comparisons yielded likelihoods stating the similarity of the current text with the knowledge available on each gene. For each type of annotation, we normalized all likelihoods for all genes (where applicable) with the highest score, so that all values were between 0 and 1. We combined all likelihoods for each gene into confidence measures (between 0 and 1) and picked the EntrezGene ID with the highest confidence score, if this was above a certain threshold. Some annotations (protein mutations and chromosomal locations), if they occur literally in the text, triggered a confidence of 1.0. This was due to the fact that such annotations were sufficient to identify a gene immediately; identical annotations for different genes/proteins were highly unlikely.

### Extracting protein-protein interactions

Our system for identifying pairs of interacting proteins built on pattern matching to find evidence for interactions, and protein identification comparable to the gene name identification described in the section above. We searched for sentences that discuss one or more PPIs using a set of language patterns learned from a large, automatically annotated corpus. For the protein identification, we construct context models from UniProt entries. Extraction of information on species is done by Ali Baba [29], based on the National Center for Biotechnology Information (NCBI) Taxonomy. We first describe our notion of language patterns and how we learn a set of these patterns without manually annotated data. We then show how evidence is found in arbitrary text, and present steps for protein name identification needed in addition to the aforementioned gene name identification.

#### Training sample

In a first step, we collect a corpus of sentences that probably describe PPIs. Therefore, we scan all of Medline. Whenever we encounter a sentence with multiple protein names, we search for every possible pair in the IntAct database of protein interactions [30]. If any of those pairs is contained in IntAct, then we add the sentence to the corpus, including the positions of the interacting proteins. Note that this step requires a sufficiently well performing protein name identification to map candidate names to (in our case, UniProt) IDs that are also referenced by IntAct. The dictionary we used for this step was different from the one provided with the BioCreative II datasets and consisted of 195,908 UniProt names and synonyms.

To avoid simple enumerations and 'random' co-occurrences of proteins, we additionally require every sentence of the corpus to contain at least one word referring to an interaction. Such words may be verbs, nouns, or adjectives typically used in the context of PPIs. Examples are verbs such as 'phosphorylates' or nouns such as 'inhibitor'. We used the word lists from Temkin and Gilder [31] and Hao and coworkers [10], adding some more.

#### Generating patterns

In the most simple case, language patterns are sequences of words that are commonly used to describe similar facts, in our case PPIs. The words of a pattern are semantically annotated to define the positions of entities (protein names), the type of a relation (cleavage, activation), and the dependency between agent and target (active and passive parts). Note that the words in a pattern need not be words as they appear in natural language, but can also be formed by linguistic tags.

Note that language patterns in our system are not regular expressions. We allow for differences between a pattern and a phrase by using sentence alignment, in the same manner as sequence alignment introduces insertions, deletions, and mismatches between DNA or protein sequences, not by introducing special characters into the pattern. Furthermore, the decision regarding whether a term of a pattern matches a term of a phrase is not binary. Instead, the specific term pair is assigned a score by looking it up in a substitution table.

#### Extraction of initial patterns

In the next step, we extract its core phrase from each sentence of the corpus. Recall that each sentence must contain one or more pairs of protein names. The core phrase of a pair of protein names is the shortest subphrase to contain both partners and the interaction word. We also add a parameterized number of tokens to the left and right. We then substitute names of proteins with their entity class. Based on the full sentence, we add those annotations described above to each position in the phrase. Each annotated core phrase now forms an initial pattern. We provide sets of these core phrases in [32].

#### From initial patterns to consensus patterns

To identify patterns that are more general than the initial ones, we perform several refinements. We first perform a pairwise alignment of every pair of initial patterns using end-space free global alignment [33] of sentences as described in the next paragraph. We considered only pairs of patterns that differed in length no more than a parameterizable threshold (*ld *in Table 3). We end up with a matrix of pair-wise similarities, called a pair-wise alignment library. We then apply UPGMA-style hierarchical clustering to the pair-wise alignment library [33]. The clustering is stopped once the similarity of the pair under consideration falls below a minimum alignment score (parameter *gl *in Table 3). This results in a set of clusters of arbitrary size, including very large and singleton clusters.

**Figure 3:**
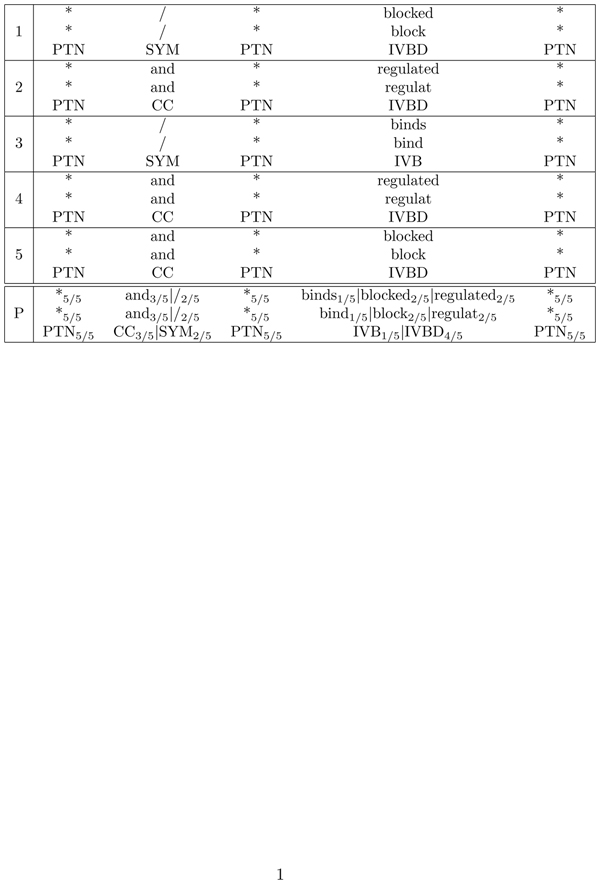
Example of a multiple sentence alignment to identify consensus patterns. Here, five patterns extracted from the corpus define one consensus pattern (bottom). Three layers are used in this example: tokens, stems, and part-of-speech (POS) tags. The weights represent the overall distributions per positions.

Next, we derive a consensus pattern for each cluster in a two-step procedure. First, we compute a multiple sentence alignment for each cluster using a greedy strategy as in progressive multiple sequence alignment algorithms such as ClustalW [35]. For every position in the multiple sequence alignment, we calculate the frequencies of observed annotations at this position. These values further influence the alignment score for the pattern aligned with unknown sentences (see Figure 3). The resulting position-specific scoring matrix forms the consensus pattern of the cluster. Figure 2 shows an example of such a motif.

#### Sentence alignment

Sentence alignment is used at two points in our system. First, initial patterns are aligned against each other to form a cluster. Second, a consensus pattern is aligned against a new sentence to extract PPIs. We only describe the latter case, because it is a generalization of the former.

The algorithm works very similarly to traditional sequence alignment. For every pair of terms, a scoring function determines the costs for this particular substitution. Sequence alignment uses scoring matrices, such as Blosum, that contain costs for every possible pair of amino acids (nucleotides), including gap penalties for inserted/deleted amino acids. In the same manner, we use scoring matrices that contain costs for term substitutions. If a term contains multiple annotations (see the following subsection), then substitution matrix must consider all of these values (tokens, stems, tags, entity classes); see below in this section.

#### Multilayer alignment

In order to generalize patterns, most often patterns and sentences become reduced to the sequence of POS and entity tags. In fact, one can think of several annotation types that together represent each term in a sequence. We see the different types of annotations as layers, and use this term in the following. The simplest form for an annotation layer is the token sequence. An alignment on the basis of tokens surely yields high precision, but this model generalizes quite poorly. Using POS tags ensures a higher recall, with slightly reduced precision. Other possible layers are the sequence of entity classes (separated from the POS tags), sentence parse information (subject and object, phrases, chunks), word stems, or lemmata.

For instance, using word stems, a switch in tense of a verb is not particularly bad if the word stem is the same ('inhibits', 'inhibited'). On the other hand, a switch in both tense and stem should be penalized ('inhibits', 'blocking'), because the meaning is (sometimes entirely) different. Thus, if a pattern uses a fixed token list for a certain position, for example 'inhibits' and 'induces', and the tense is fixed as well (only present tense), a sentence containing 'inhibited' in past tense still achieves a good score, because at least the word stem 'inhibit' is valid. For our experiments, we use tokens, word stems, tags, and entity classes to annotate terms. For POS tags and entity classes, we use a heuristic substitution matrix to score about 50 different tags against each other. For tokens and word stems, matches yield +1, replacements -1, and gaps -3.

We calculate the multilayer alignment exactly like single-layer alignments. The only difference is that this scoring function considers not only one substitution matrix, but several. For each layer, there is a dedicated substitution matrix. An example for a multilayer alignment that produces a consensus patterns is shown in Figure 3.

#### Pattern matching

For matching patterns against texts, we first restricted texts to sentences that contain at least two proteins. The matching was then done using the aforementioned multilayer alignment of patterns with similarly represented sentences (POS tags/proteins, tokens, and word stems). We precalculated the maximum score that could be achieved with each pattern by aligning it to itself. The fraction of the alignment score of patterns and sentence and this maximum score then defined the quality of a match. A parameterizable threshold decides whether the match is good enough and the sentence is likely to express a PPI. When a pattern matched a sentence, this also defined the exact positions of all proteins involved. These names were then taken to identify the actual proteins.

#### Identification of proteins

Once evidence for a PPI was extracted, each protein also had to be identified. Like in the gene mention normalization, the named entity recognition resulted in a set of potential candidate IDs for each name. Identification also was done as described above. We further narrowed down the list of potential candidates by requiring both proteins of a given interaction to originate from the same species. This comparison on both sets was done on the plain UniProt accession code that includes the species (for instance, 'FADD_HUMAN'). We treated all *Saccharomyces *and *Schizosaccharomyces *as yeast in general. Once both sets had been narrowed down, we applied a parameterizable heuristic to identify the correct species. We checked the abstract for any species names first; if no species fitting the candidate set was found, then we took the first human/mouse/yeast variant from the set (if any), or picked one at random (parameter *sr *in Table 3). Species names in the abstract (mapped to NCBI taxonomy IDs by Ali Baba [29]) were compared with the controlled vocabulary provided by SwissProt/ExPASy [36]. In cases in which multiple IDs per protein remained that had similiar likelihoods assigned by the disambiguation component, we considered sending not only the best scored candidate but multiple (parameter *ids *in Table 3). Also, we used heuristics to pick a protein if multiple species were possible ('FADD_HUMAN', 'FADD_MOUSE', and 'FADD_RAT'); pick the (first) species mentioned together with the protein in the abstract, pick human, mouse, yeast, or the species of the candidate with the best score. This order was determined by parameter *sr *in Table 3.

#### Filtering false positives/unimportant interactions

We noticed that some mentions of PPIs were missing from the gold standard, presumably because they did not form the main thrust of a publication, but were rather introductory and for better understanding of the overall setup. This was particularly true for PPIs mentioned in the introduction of an article, but never again later. Also, we found some PPIs in dangling text and references, which should not be reported. We filtered pairs of interacting proteins that occurred too few times in a single article (parameter *m *in Table 3). The gold standard demanded clear evidence for physical interactions of proteins. We treated PPIs mentioned together with any reference to large scale assays as false positives; this was indicated by terms such as 'assay', 'yeast 2-hybrid', 'beads', 'prey', 'traits', 'biochemical', and 'in vitro'. However, the respective PPI could occur in the same text multiple times, without such hints, and thus be retained.

## Availability

Our methods for gene, protein, and species identification, and PPIs are available as part of the BioCreative Meta Services; see the overview presented in this supplement [6].

## Abbreviations

DMAP, direct memory access parsing; GO, Gene Ontology; NCBI, National Center for Biotechnology Information; IPS, interaction pair subtask; POS, part-of-speech; PPI, protein-protein interaction; UPGMA, unweighted pair group method with aritmetic mean.

## Competing interests

The authors declare that they have no competing interests.

## Authors' contributions

JH, CP, LR, and HS designed and implemented the gene normalization system. JH designed and implemented the IPS system. UL guided research on the IPS project. MS guided research on the gene normalization project. JH, MS, and CP wrote the manuscript. All authors have read and approved the final manuscript.

## Additional data files

The following additional data are available with the online version of this paper. Additional data file 1 provides the regular expression of false-positive gene names. Additional data file 2 provides the verbs, nouns, adjectives referring to protein-protein interactions.

## Supplementary Material

Additional file 1 Regular expressions catching gene names that are too unspecific - that is, false positives in the BioCreative II gene normalization data. These result, for example, from protein families, tissues, or other 'spurious' synonyms in the original lexicon. Click here for file

Additional file 2 The file contains verbs, nouns, and adjectives that can refer to PPIs. Click here for file
